# Risk of Fracture During Androgen Deprivation Therapy Among Patients With Prostate Cancer: A Systematic Review and Meta-Analysis of Cohort Studies

**DOI:** 10.3389/fphar.2021.652979

**Published:** 2021-08-06

**Authors:** Cheng Chih Wu, Po Yen Chen, Shih Wei Wang, Meng Hsuan Tsai, Yu Chin Lily Wang, Ching Ling Tai, Hao Lun Luo, Hung-Jen Wang, Chung Yu Chen

**Affiliations:** ^1^ Department of Pharmacy, Kaohsiung Chang Gung Memorial Hospital, Kaohsiung, Taiwan; ^2^ School of Pharmacy, Kaohsiung Medical University, Kaohsiung, Taiwan; ^3^ Division of Urology, Kaohsiung Chang Gung Memorial Hospital, Kaohsiung, Taiwan; ^4^ Chang Gung University College of Medicine, Kaohsiung, Taiwan; ^5^ Master Program in Clinical Pharmacy, School of Pharmacy, Kaohsiung Medical University, Kaohsiung, Taiwan; ^6^ Department of Pharmacy, Kaohsiung Medical University Hospital, Kaohsiung, Taiwan; ^7^ Department of Medical Research, Kaohsiung Medical University Hospital, Kaohsiung, Taiwan; ^8^ Center for Big Data Research, Kaohsiung Medical University, Kaohsiung, Taiwan

**Keywords:** fracture, prostate cancer, antiandrogen, androgen deprivation therapy, luteinizing hormone-releasing hormone agonist

## Abstract

**Background:** Androgen deprivation therapy (ADT) suppresses the production of androgen, and ADT is broadly used for intermediate or higher risk disease including advanced and metastatic cancer. ADT is associated with numerous adverse effects derived from the pharmacological properties. Previous meta-analysis on fracture risk among ADT users possessed limited data without further subgroup analysis. Risk estimation of updated real-world evidence on ADT-related fracture remains unknown.

**Objectives:** To assess the risk of fracture and fracture requiring hospitalization associated with ADT among prostate cancer population on different disease conditions, treatment regimen, dosage level, fracture sites.

**Methods:** The Cochrane Library, PubMed, and Embase databases were systematically screened for eligible cohort studies published from inception to March 2020. Two authors independently reviewed all the included studies. The risks of any fracture and of fracture requiring hospitalization were assessed using a random-effects model, following by leave-one-out, stratified, and sensitivity analyses. The Grading of Recommendations Assessments, Development and Evaluations (GRADE) system was used to grade the certainty of evidence.

**Results:** Sixteen eligible studies were included, and total population was 519,168 men. ADT use is associated with increasing fracture risk (OR, 1.39; 95% CI, 1.26–1.52) and fracture requiring hospitalization (OR, 1.55; 95% CI, 1.29–1.88). Stratified analysis revealed that high-dose ADT results in an elevated risk of fracture with little statistical heterogeneity, whereas sensitivity analysis restricted to adjust for additional factors indicated increased fracture risks for patients with unknown stage prostate cancer or with no restriction on age with minimal heterogeneity. The GRADE level of evidence was moderate for any fracture and low for fracture requiring hospitalization.

**Conclusion:** Cumulative evidence supports the association of elevated fracture risk with ADT among patients with prostate cancer, including those with different disease conditions, treatment regimens, dose levels, and fracture sites. Further prospective trials with intact information on potential risk factors on fracture under ADT use are warranted to identify the risky population.

## Introduction

Prostate cancer is the most common cancer among men, and remains the second leading cause of death in the United States in 2020 (Siegel et al., 2020). Standard treatments based on health status for localized or locally advanced disease including watchful waiting, active surveillance, radical prostatectomy (RP), and radiotherapy (RT) with or without androgen deprivation therapy (ADT) (Gamat and McNeel, 2017; NCCN, 2020). For hormone-sensitive and castration-resistant metastatic disease, second-generation antiandrogens, docetaxel, radium-223, sipuleucel-T, and poly ADP ribose polymerase (PARP) inhibitors are the treatment options for various patient groups.

ADT is an effective treatment for prostate cancer (Gamat and McNeel, 2017; NCCN, 2020), including for those patients receiving RT or RP adjuvant therapy for localized or locally advanced disease or with castration-resistant or hormone-sensitive metastatic disease as well as patients with contraindications for or intolerance of radical treatment in very high-risk groups (A M El Batri et al., 2019), thus improving progression-free, overall survival (Tosco et al., 2019). Orchiectomy is a method of surgical castration, whereas luteinizing hormone–releasing hormone (LHRH) agonists, LHRH antagonists, and antiandrogen are used for chemical castration (NCCN, 2020). However, the long-term risks involved with ADT require further study.

Fractures are an important public health issue resulting in a heavy health care burden and affecting an individual’s quality of life (Santini et al., 2020). Furthermore, fracture among prostate cancer patients remains an independent negative predictor of overall survival (Oefelein et al., 2002). Research reported that ADT was significantly associated with reduced bone mineral density (BMD) in the lumbar spine, femoral neck, and total hip compared with controls (Kim et al., 2019). Nevertheless, BMD can only explain 60–80% of bone variation, and T-score is lack of complete insight on bone quality and construction (Li et al., 2004; Fitton et al., 2015). The association between fracture risk and ADT has been investigated in previous meta-analyses; however, some studies have not performed subgroup analysis based on disease state of prostate cancer, thus rendering indistinct the risks of pathological and nonpathological ADT-related fractures (Serpa Neto et al., 2010).

Because of the limited predictive validity of BMD values on fracture and incomplete data from previous meta-analyses, more comprehensive systematic reviews are warranted to fill this knowledge gap. Therefore, we quantitatively assessed all eligible studies focusing on the effect of ADT on fracture risk to obtain complete information.

## Methods

This systematic review was conducted in accordance with the Preferred Reporting Items for Systematic Reviews and Meta-Analyses (PRISMA; Supplementary Material S1) and Meta-analysis of Observational Studies in Epidemiology (MOOSE; Supplementary Material S2) (Stroup et al., 2008; Moher et al., 2009) guidelines and registered in PROSPERO (CRD42020197561).

### PICO Question

We followed the participants(P), intervention(I), comparators(C), and outcomes(O) for study selection under the PICO framework. The proposed clinical question of the present systematic review and meta-analysis is as followed: does having used ADT (compared to no use) increase the risk of fracture among prostate cancer population (P: prostate cancer population; I: ADT use; C: no ADT use; O: fracture or fracture requiring hospitalization)?

### Search Strategies and Study Selection

We performed a comprehensive literature search of the Cochrane Library, PubMed, and Embase databases. The searching process has been conducted on 24th, April 2020. Literature reviewed was dated from inception through March 2020. The following search query, comprising a combination of keywords, was used in the 3 databases derived from previous PICO question: “prostate cancer AND (androgen deprivation OR androgen suppression OR chemical castration OR antiandrogen OR gonadotropin-releasing hormone agonist) AND fracture” (Supplementary Material S3). Two independent researchers reviewed the titles and abstracts to determine the eligibility of the articles, and subsequently read the full-text of the eligible articles.

### Included and Excluded Criteria

Studies had to be cohort studies published in English and from all countries. Hazard ratio (HR), odds ratio (OR), or relative risk (RR) along with corresponding 95% confidence interval (CI) were extracted. If the study population was limited to patients with localized prostate cancer, we grouped the results as pertaining to localized prostate cancer. We considered the results of studies without a clear delineation of prostate cancer stage or metastatic prostate cancer to pertain to unknown stage prostate cancer. If the results of multiple included studies were derived from the same database, we selected the study with the longest duration. If there was lacking of primary outcome on total fracture risk, stratified results from separate dosage intensities were combined. We excluded researches that focused on the risk of BMD change and osteoporosis among ADT users. Studies lacking any requisite data were also excluded.

### Data Extraction and Quality Assessment

Two authors independently used a self-developed form to record data. The form documented the study source, study design, patient characteristics, exposure assessment, treatment characteristics, outcome categories, outcome measures, definitions of prostate cancer and fracture, adjusted factors, and conflicts of interest. Discrepancies regarding these data were resolved through consensus.

The same authors assessed quality using the Newcastle-Ottawa scale (NOS) and Grading of Recommendations Assessments, Development and Evaluations (GRADE) assessment. The NOS is used to assess methodological quality in observational studies and is a validated 8-item tool that characterizes participant selection (4 items), comparability of populations (1 item), and outcome assessment (3 items). Low quality was defined as an NOS score of <7, and high quality was defined as an NOS score of ≥7 (Wells et al., 2021). Moreover, GRADE approach was applied for assessing the overall certainty of evidence from the included studies (Guyatt et al., 2011).

### Data Synthesis and Statistical Analysis

The primary outcome was any fracture risk under ADT use. Leave-one-out sensitivity analysis was used to determine the robustness of the overall findings through sequential elimination of each trial. Stratified analyses were conducted based on disease condition, treatment regimen, dosage, age, and fracture site to identify relevant subgroups and investigate potential sources of heterogeneity among the studies. Sensitivity analysis was used to exclude studies with ≤4 adjusting factors for stratified groups comprising >3 studies. Secondary analyses were performed to evaluate the risk of fracture requiring hospitalization from ADT. Analyses were performed with Review Manager (version 5.4.1 for Windows, Cochrane Collaboration, Copenhagen, Denmark, 2014). Dichotomous outcomes are reported as ORs, and time-to-event outcomes are reported as HRs, each with a 95% CI. HRs and ORs were determined through the inverse variance method. Because the absolute risk of fracture is low, RRs or HRs from cohort studies were used to estimate the OR (Zhou et al., 2016).

Fixed- or random-effects models were applied on calculating effect size and 95% CI on the concept of between-study heterogeneity. The heterogeneity derived from separated studies was quantified using *I* ^2^ test. Forest plots were constructed to evaluate statistical heterogeneity. Statistic heterogeneity was assessed for each outcome with the *I* ^2^ method. We considered *I* ^2^ values of 25–49%, 50–74%, and ≥75% to represent low, moderate, and high heterogeneity, respectively (Higgins et al., 2003). When *I* ^2^ was higher than 50%, random-effects model was chosen; when it was lower than 50%, fixed-effects model was preferred. All *p* values were 2 sided, and statistical significance was set at *p* < 0.05. A funnel plot was constructed to assess bias visually, and the Egger test was performed to evaluate the asymmetry in the funnel plot. The trim-and-fill method was used to estimate and adjust for potential effects from unpublished studies. Publication bias, analyzed using Comprehensive Meta-Analysis (Version 3, Biostat, Englewood, NJ, United States) was examined only when more than 10 studies were included in the analysis of the primary outcomes.

## Results

### Search Results and Characteristics of Included Studies

**FIGURE 1:**
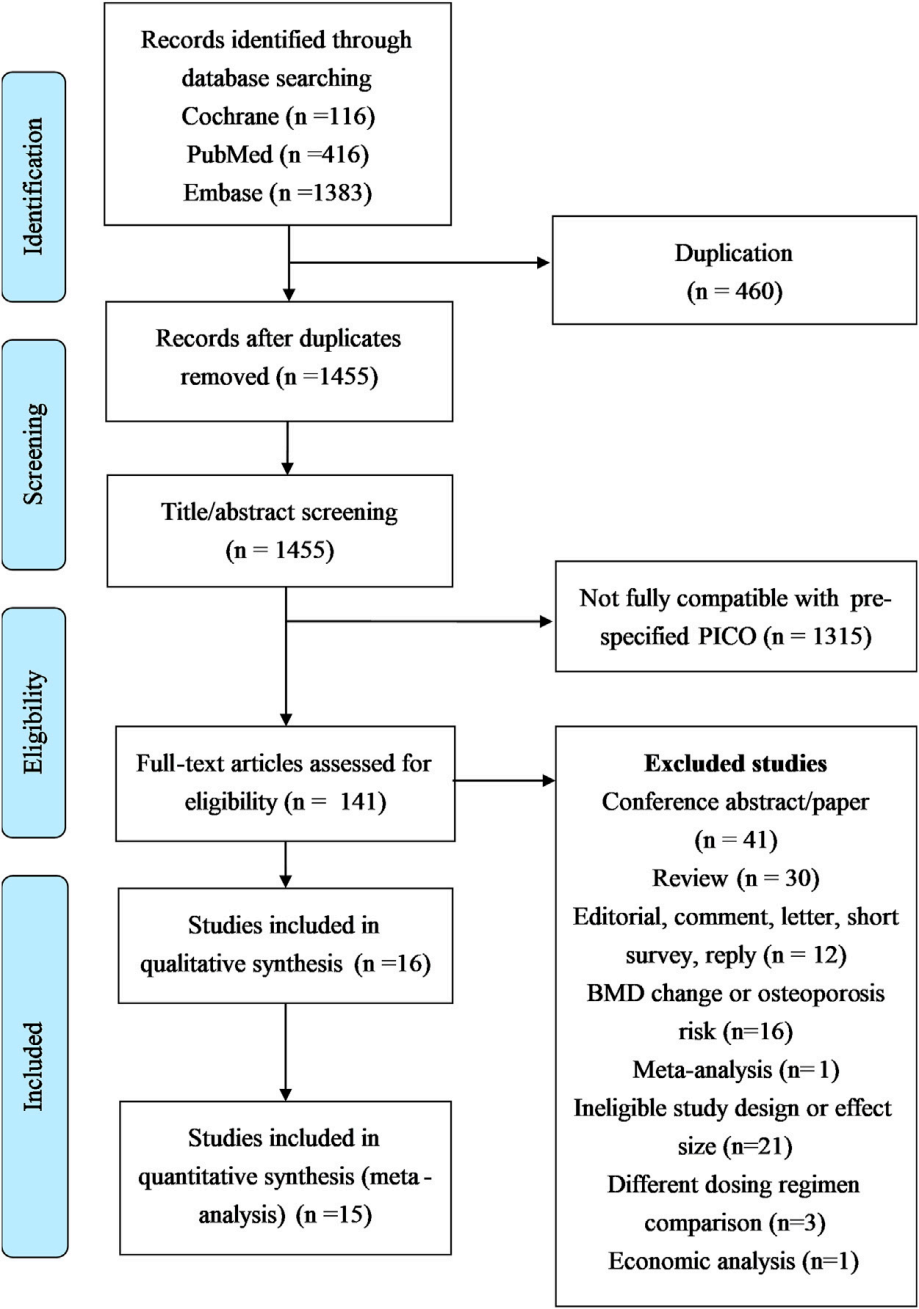
Preferred reporting items for systematic reviews and meta-analyses (PRISMA) flowchart.

Figure 1 delineates the search protocol, which yielded 1915 articles initially: 416 from PubMed, 1,383 from Embase, and 116 from the Cochrane Library. Of these, 460 duplicated articles were initially excluded. Thereafter, we excluded 1,314 studies because of their incompatibility with the specified participants, interventions, comparisons, and outcomes screening in this study. In the last, we identified 141 articles which are considered closely related to the present study objectives. Of these 141 studies, 41 were excluded because they were conference abstracts/papers; 30 were excluded because they were review articles; 12 were excluded because they were editorials, comments, letters, short surveys, or replies; 24 were excluded because they had ineligible study designs or effect sizes or different dosing regimens; 1 was excluded because it was a published meta-analysis including the risks of fracture, osteoporosis, and osteopenia with ADT (Serpa Neto et al., 2010). Finally, 16 full-text studies (*n*  = 519,168 individuals) (López et al., 2005; Shahinian et al., 2005; Smith et al., 2005; Smith et al., 2006; Alibhai et al., 2010; Beebe-Dimmer et al., 2012; Shao et al., 2013; Kaipia et al., 2014; Morgans et al., 2014; Teoh et al., 2015; Wang et al., 2015; Wu et al., 2015; Wallis et al., 2016; Lee et al., 2017; Nguyen et al., 2018; Wallander et al., 2019) were included.

The characteristics of the 16 studies included in the meta-analysis are summarized in Table 1. These articles were published from 2005 to 2019. Three were conducted in Europe [Sweden (Wallander et al., 2019), Finland (Kaipia et al., 2014), and Spain (López et al., 2005)], 8 in the United States (Shahinian et al., 2005; Smith et al., 2005; Smith et al., 2006; Beebe-Dimmer et al., 2012; Shao et al., 2013; Morgans et al., 2014; Wallis et al., 2016; Nguyen et al., 2018), 1 in Canada (Alibhai et al., 2010), and 4 in Asia [Taiwan (Wu et al., 2015), New Zealand (Wang et al., 2015), and China (Teoh et al., 2015; Lee et al., 2017)]. Thirteen studies assessed the risk of any fracture (López et al., 2005; Shahinian et al., 2005; Smith et al., 2005; Smith et al., 2006; Alibhai et al., 2010; Shao et al., 2013; Kaipia et al., 2014; Morgans et al., 2014; Teoh et al., 2015; Wu et al., 2015; Lee et al., 2017; Nguyen et al., 2018; Wallander et al., 2019), 5 assessed the risk of fracture requiring hospitalization (Shahinian et al., 2005; Beebe-Dimmer et al., 2012; Wang et al., 2015; Wallis et al., 2016; Lee et al., 2017), and 3 assessed the risk of hip fracture (Smith et al., 2006; Kaipia et al., 2014; Wallander et al., 2019). The number of study participants ranged from 201,797 in the Surveillance, Epidemiology, and End Results (SEER) program to 452 in a single-hospital study from China. Most included studies reported HRs (Smith et al., 2005; Alibhai et al., 2010; Beebe-Dimmer et al., 2012; Shao et al., 2013; Teoh et al., 2015; Wu et al., 2015; Wallis et al., 2016; Lee et al., 2017; Nguyen et al., 2018; Wallander et al., 2019), 4 reported ORs (Smith et al., 2006; Kaipia et al., 2014; Morgans et al., 2014; Wang et al., 2015), and 2 reported RRs (López et al., 2005; Shahinian et al., 2005). Nine studies considered patients with localized prostate cancer as their target demographic (Smith et al., 2005; Smith et al., 2006; Alibhai et al., 2010; Beebe-Dimmer et al., 2012; Shao et al., 2013; Morgans et al., 2014; Teoh et al., 2015; Wang et al., 2015; Wallis et al., 2016), but the other studies lacked prespecified criteria. Eight studies involved individuals aged only ≥66 years (Shahinian et al., 2005; Smith et al., 2005; Alibhai et al., 2010; Beebe-Dimmer et al., 2012; Shao et al., 2013; Wallis et al., 2016; Nguyen et al., 2018; Wallander et al., 2019), but no age restriction was applied in the remaining studies. Five studies were derived from the SEER program (Shahinian et al., 2005; Beebe-Dimmer et al., 2012; Shao et al., 2013; Wallis et al., 2016; Nguyen et al., 2018), from 1992 through either 1997 (Shahinian et al., 2005) or 2010 (Nguyen et al., 2018). ADT was verified from administrative or claims data in 14 studies (López et al., 2005; Shahinian et al., 2005; Smith et al., 2005; Smith et al., 2006; Abrahamsen et al., 2007; Lau et al., 2009; Alibhai et al., 2010; Beebe-Dimmer et al., 2012; Shao et al., 2013; Wang et al., 2015; Wu et al., 2015; Wallis et al., 2016; Nguyen et al., 2018; Wallander et al., 2019); however, some studies used medical records (Kaipia et al., 2014; Teoh et al., 2015; Lee et al., 2017), and one used patient reports (Morgans et al., 2014). Potential confounding factors were matched or adjusted for in individual studies. Authors from 6 studies (Smith et al., 2005; Smith et al., 2006; Shao et al., 2013; Morgans et al., 2014; Teoh et al., 2015; Wallander et al., 2019) declared a conflict of interest, whereas the others reported no conflict of interest.

**TABLE 1:** Characteristics of studies included in meta-analysis.

Study, publication year, location	Study period	Ages studied (y)	Study size	Study source	Exposure assessment	RT/RP documentation	Category of ADT	Antiresorptive medication use	Outcome categories	Fracture definition	Adjusted factors	Conflict of interest
Wallander et al. (2019), Sweden	2008–2014	>65; Prostate cancer with ADT: 82 ± 7; Prostate cancer without ADT: 79 ± 7.4	20,082	Fractures and fall injuries in the elderly cohort (FRAILCO)	No information	No information	LHRH agonist	Yes	Fracture, hip fracture, major osteoporotic fracture	*ICD-10-CM* codes S720-S722 + surgical procedure code	Age, height, weight, previous fracture, glucocorticoid use, rheumatoid arthritis, estimated overconsumption of alcohol, secondary osteoporosis, CCI, alendronate, calcium + vitamin D, previous known fall	Yes
Nguyen et al. (2018), USA	1992–2009	>66	117,962	SEER Program	Administrative or claims data (Healthcare Common Procedure Coding System, Common Procedure Terminology)	RT and RP included (RT adjustment)	LHRH agonists or antagonists	No	Fracture	*ICD-9-CM* codes 733.1x, 800–829	Age, race/ethnicity, marital status, urban or rural location, SEER geographic area, year of diagnosis, socioeconomic status, comorbidity score, cancer stage at diagnosis, Gleason score, RT, surgery	No
Lee et al. (2017), China	2001–2011	72.9 ± 8.5	741	Queen Mary Hospital (single center)	Medical records	Unknown	LHRH agonists or antagonists, antiandrogen	Yes	Hospital admission for fracture	*ICD-9-CM* codes 733, 805–809, 810–819, 820–829	Age, DM, ADT	No
Wallis et al. (2016), Canada	2000–2008	>65	60,156	SEER program	Administrative or claims data	Include RT and RP	LHRH agonists or antagonists, bilateral orchiectomy	Unknown	Fracture requiring hospitalization	*ICD-9-CM* codes 733.1x, 800–829	Age, grade, race, marital status, CCI, prediagnosis history of osteoporosis	No
Wang et al. (2015), New Zealand	2004–2012	68 ± 9.4	25,544	New Zealand Cancer Registry	Administrative or claims data (PharmaceuticalCollection)	Unknown	LHRH agonist, antiandrogen, LHRH agonist + antiandrogen, bilateral orchiectomy, bilateral orchiectomy + pharmacologic therapy (LHRH agonist ± antiandrogen)	Yes	Fracture (any fracture, hip fracture) requiring hospitalization	*ICD-10-CM* codes S12, S22, S32, S42, S52, S62, S72, S82, S92	Age, ethnicity	No
Teoh et al. (2015), China	2000–2009	69.5 ± 6.5	452	Chinese University of Hong Kong (single center)	Medical records	Post RT or RP	LHRH agonist, bilateral orchiectomy, bilateral orchiectomy + LHRH agonist	Unknown	Fracture	Not mentioned	Age, DM, hypertension, hyperlipidemia, preexisting ischemic heart disease, ECOG performance status	No
Wu et al. (2015), Taiwan	1998–2007	No restriction	17,359	National Health Insurance Research Database	Administrative or claims data	Include RT and RP	LHRH agonist, bilateral orchiectomy	No	Fracture	*ICD-9-CM* codes 733.1x, 800–827	Age, socioeconomic status, CCI, other cancer treatments received	No
Morgans et al. (2014), USA	1994–1995	Enrolled 39-89 years	843	Prostate Cancer Outcomes Study (enrolled as part of SEER program)	Patient report	Include RT and RP	LHRH agonist, antiandrogen, LHRH agonist + antiandrogen	Yes	Fracture	Patient self-reports	ADT treatment, age, race, marital status, CCI, Gleason score	Yes
Kaipia et al. (2014), Finland	1998–2008	ADT group: 69.5 ± 6.5; Non-ADT group: 68.2 ± 5.9	6,051	Tampere University Central Hospital database	Medical records	Include RT and RP	LHRH agonist, bilateral orchiectomy	No	Hip fracture	*ICD-10-CM* codes S72.0-S72.2	Age, PSA level (outcome: low risk, medium risk, high risk)	No
Shao et al. (2013), USA	1992–2007	>65	75,994	SEER program	Administrative or claims data	Include RT and RP	LHRH agonist, bilateral orchiectomy	Yes	Fracture	*ICD-9-CM* codes 733.1x, 800–829	Age, year of diagnosis, race, tumor grade and stage, risk factor index, comorbidity, cumulative dose of LHRH agonists in 1-month equivalent doses	Yes
Beebe-Dimmer et al. (2012), USA	1996–2003	>65	80,844	SEER program	Administrative or claims data	Include RT and RP (adjustment)	LHRH agonist, bilateral orchiectomy	No	Fracture, fracture requiring hospitalization	*ICD-9-CM* codes 733.1x, 800–829	Age at prostate cancer diagnosis, race, tumor grade, clinical T stage, comorbidity, history of fracture, osteoporosis or osteopenia prior to prostate cancer diagnosis, and primary treatment	Yes
Alibhai et al. (2010), Canada	1995–2005	>66	38,158	Ontario Cancer Registry records	Administrative or claims data	Include RP	LHRH agonist, antiandrogen, LHRH agonist + antiandrogen, bilateral orchiectomy	Yes	Fracture, fragility fracture	Hospital inpatient and outpatient claims	Age, prior bone thinning medication, prior chronic kidney disease, prior dementia, prior fragility fracture, prior osteoporosis diagnosis or treatment, prior rheumatologic disease, regular primary care access	No
Smith et al. (2006), USA	1998–2003	LHRH agonist: 73.4 ± 8.3; Non-LHRH agonist: 68.9 ± 9.0	12,120	Medical claims from 16 large American companies	Administrative or claims data	Unknown	LHRH agonist	No	Fracture hip fracture,vertebral fracture	*ICD-9-CM* codes 767, 800–839, 850–854, V66.4s	LHRH agonist treatment, age, comorbidity, income, observation period, geographic region, health plan type	Yes
Smith et al. (2005), USA	1992–1994	>65	11,661	Medicare public use file databases	Administrative or claims data	Unknown	LHRH agonist	No	Fracture	*ICD-9-CM* codes 733.1–733.19, 22,325–22,327, 805, 806, 820–829	Age, race, location, cardiovascular disease, DM, LHRH agonist treatment, duration of LHRH agonist treatment	Yes
Shahinian et al. (2005), USA	1992–1997	>65	50,613	SEER program	Administrative or claims data	RT and RP included (adjustment)	LHRH agonist, bilateral orchiectomy	No	Fracture, fracture requiring hospitalization	*ICD-9-CM* codes 733.1x, 800–829	Age; race/ethnicity; SEER region, grade of prostate cancer; cancer stage; year of diagnosis; level of education; income; CCI; number of provider visits within the 12 months before diagnosis; presence of osteoporosis, osteopenia, or fracture within the 12 months before diagnosis; presence or absence of treatment with RP or RT	No information
López et al. (2005), Spain	No information	ADT groups: 72 ± 7; Control group: 70 ± 8	588	Urology and pathology departments	Administrative or claims data	Unknown	LHRH agonists ± androgen receptor blockers	No	Fracture	Medical records	Age, previous fracture, smoking habits, alcohol habits	No

Abbreviations: ADT, androgen deprivation therapy; CCI, Charlson comorbidity index; DM, diabetes mellitus;ICD-9-CM, International Classification of Diseases, Ninth Revision, Clinical Modification; ICD-10-CM, International Classification of Diseases, Tenth Revision, Clinical Modification; LHRH, luteinizing hormone–releasing hormone; PSA, prostate-specific antigen; RP, radical prostatectomy; RT, radiotherapy; SEER, Surveillance, Epidemiology, and End Results; SD, standard deviation.

### Main Analysis

**FIGURE 2:**
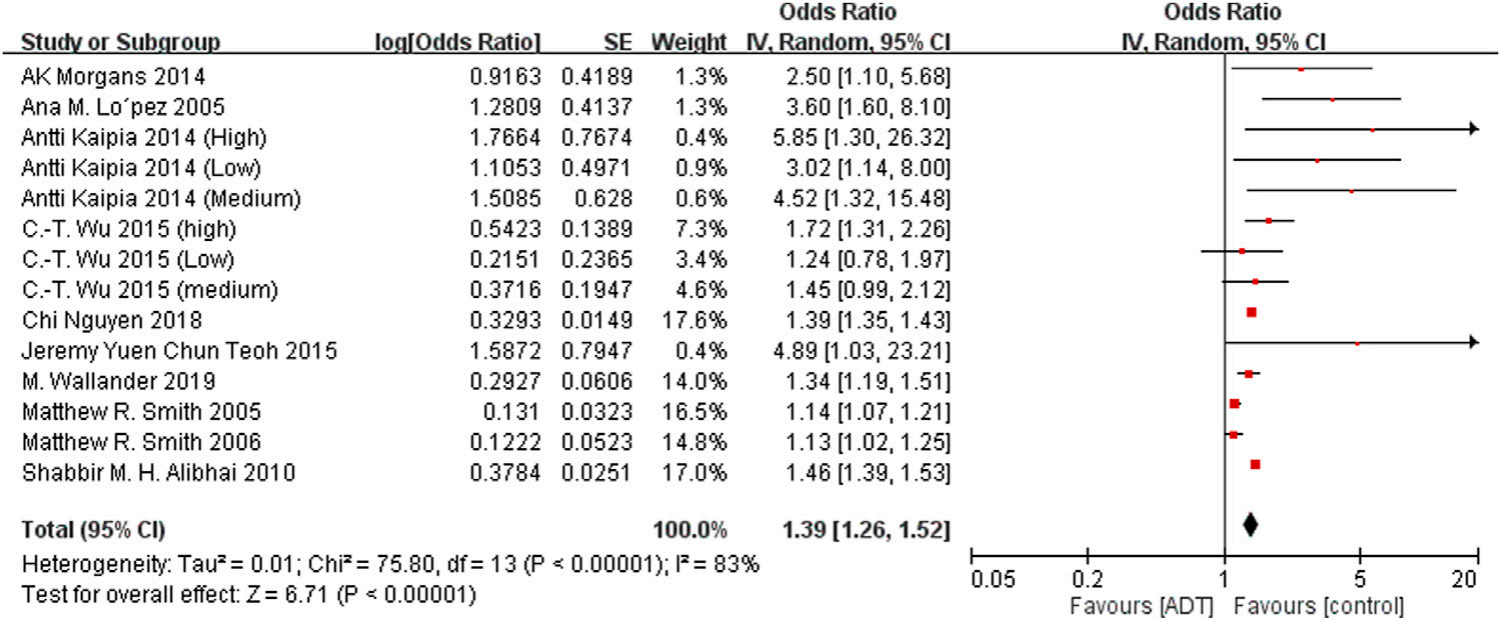
Forest plot for androgen deprivation therapy and risk of fracture.

**FIGURE 3:**
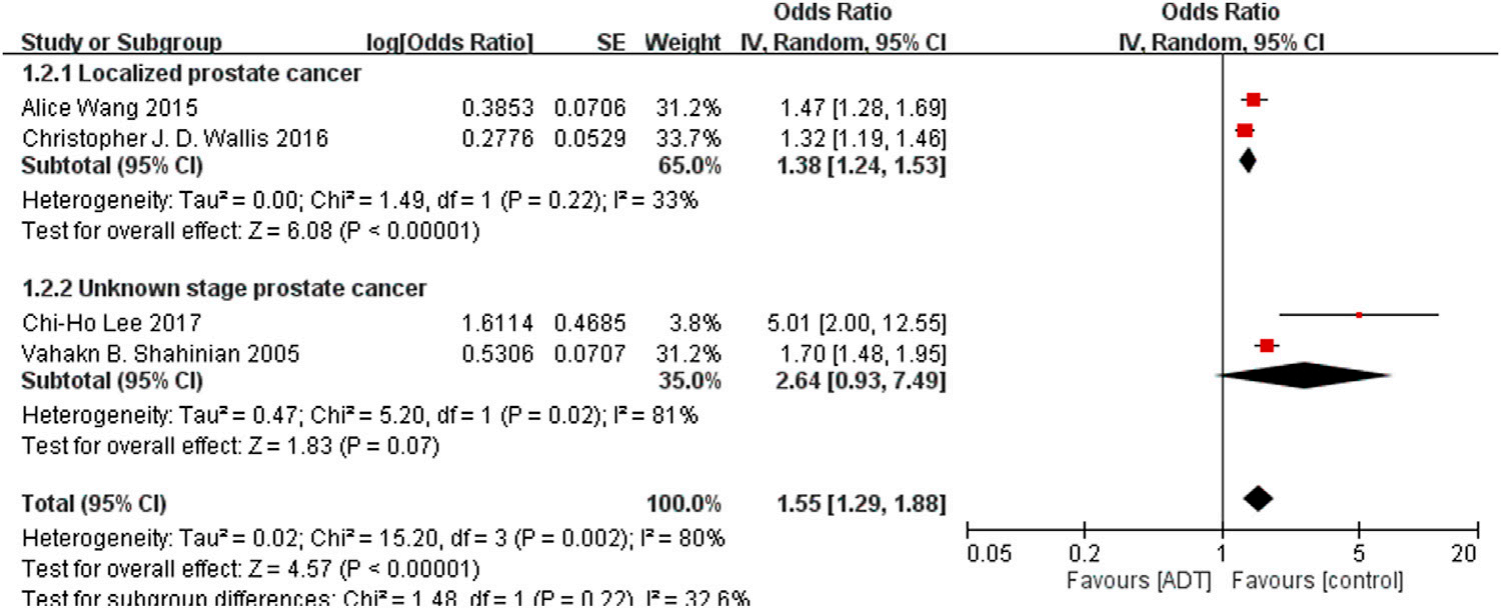
Forest plot for androgen deprivation therapy and risk of fracture requiring hospitalization.

The main analysis investigated the association of ADT with any fracture risk and risk of fracture requiring hospitalization. Among the included studies, 13 investigated any fracture risk. With the results of 3 eligible studies derived from the same database (Wallis et al., 2016; Shao et al., 2013; López et al., 2005)excluded, ADT was associated with an increased risk of any fracture (OR = 1.39; 95% CI, 1.26–1.52; *I* ^2^ = 83%), and this result was accompanied by significant heterogeneity (Figure 2). After removal of study from Beebe-Dimmer et al. (2012) from same data source with shorten researching date, secondary analyses found increased risk of fracture requiring hospitalization from ADT (OR = 1.55; 95% CI, 1.29–1.88; *I* ^2^ = 80%; Figure 3).

### Stratified and Sensitivity Analyses

Because of the high heterogeneity in the main analysis, stratified analysis of the numerous treatment methods and clinical factors was used to explore any further potential effects. Leave-one-out sensitivity analysis was employed for the primary outcome, and no single study substantially influenced the pooled OR, indicating that the present results remained stable (Supplementary Material S4). Furthermore, we conducted stratified analysis based on clinical parameters. First, we performed stratified analysis for the primary outcome in terms of disease condition. The risk of any fracture was elevated among individuals with localized (OR = 1.30; 95% CI, 1.08–1.57; *I* ^2^ = 92%) or unknown stage prostate cancer (OR = 1.51; 95% CI, 1.32–1.73; *I* ^2^ = 55%), with high heterogeneity. Diminished heterogeneity was observed among individuals with fracture risk with unknown stage prostate cancer under the restriction that studies must adjust for ≥4 factors (OR = 1.39; 95% CI, 1.35–1.43; I^2^ = 0%); however, all studies focusing on the patients with localized prostate cancer have been adjusted for ≥4 factors without further conduction of sensitivity analysis. Second, we explored the effects irrespective treatment regimen. Stratified analysis of LHRH agonist revealed an elevated fracture risk with limited information on antiandrogen or orchiectomy. In sensitivity analysis, the fracture risk among patients treated with LHRH agonists remained consistent, without a significant reduction in heterogeneity (stratified analysis: *I* ^2^ = 66%; sensitivity analysis: *I* ^2^ = 67%). Third, stratified analyses indicated an increase in fracture risk with increased dosage, and the ORs for low, medium, and high dosage were 1.08, 1.20, and 1.54, respectively; all three dose groups exhibited low heterogeneity (*I* ^2^ = 0%). No sensitivity analysis was conducted for this stratification because all eligible studies were adjusted for more than 4 factors. Fourth, we analyzed only those individuals aged >65 years (OR = 1.34; 95% CI, 1.22–1.46; *I* ^2^ = 87%) and a group with no age restriction (OR = 3.40; 95% CI, 2.07–5.58; *I* ^2^ = 0%). Elevation in heterogeneity was noted on >65-year-old population (stratified analysis: *I* ^2^ = 87%, sensitivity analysis: *I* ^2^ = 92%) while lower heterogeneity has been noted on the population without age restriction (*I* ^2^ = 0%). Finally, we restricted the fracture risk upon ADT administration to only that of the hip and discovered an elevated fracture risk (OR = 1.43; 95% CI, 1.10–1.86) with consistently elevated heterogeneity (Table 2).

**TABLE 2:** Stratified and Sensitivity analyses of Primary Outcomes.

	Stratified analysis			Sensitivity analysis (conduced on ≥3 studies of each stratified analysis)		
Factor	No. of studies	Summary of adjusted OR (95% CI)	Heterogeneity, I^2^ (%)	No. of studies (>4 adjusted factors)	Summary of adjusted OR (95% CI)	Heterogeneity, *I* ^2^ (%)
Disease condition						
Localized	5	1.30 (1.08, 1.57)	92	-	-	-
Unknown stage	5	1.51 (1.32, 1.73)	55	2	1.39 (1.35, 1.43)	0
Treatment regimen						
LHRH agonist	4	1.26 (1.13, 1.40)	66	3	1.19 (1.08, 1.31)	67
Antiandrogen	-	-	-	-	-	-
Orchiectomy	-	-	-	-	-	-
Dose level						
Low	2	1.08 (1.02, 1.15)	0	-	-	-
Medium	2	1.20 (1.08, 1.32)	0	-	-	-
High	2	1.54 (1.45, 1.63)	0	-	-	-
Age						
Not restricted	3	3.40 (2.07, 5.58)	0	2	2.89 (1.40, 5.98)	0
>65 years only	7	1.34 (1.22, 1.46)	87	5	1.29 (1.17, 1.42)	92
Fracture site						
Hip	3	1.43 (1.10, 1.86)	75	2	1.23 (1.04, 1.45)	78

Abbreviations: LHRH, luteinizing hormone–releasing hormone; OR, odds ratio.

### Quality of Included Studies, GRADE Assessment, and Publication Bias

**FIGURE 4:**
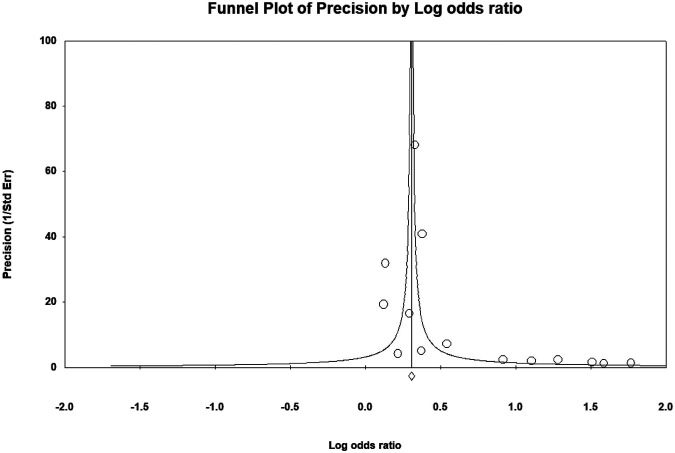
Funnel plot for assessing publication bias.

Quality assessment of the cohort studies revealed that 6 studies had low quality (6 points) and 10 studies had high quality (7–9 points; Supplementary Material S5). The certainty of evidence for the risk of any fracture during ADT administration was moderate, whereas the certainty of evidence for fracture requiring hospitalization was low (Table 3). Serious risk of bias and inconsistency were noted, but indirectness and imprecision were not severe. A major discrepancy among the primary and secondary outcome was observed regarding that ADT only exhibited a dose-response relationship with the risk of any fracture. Publication bias was not suspected in the present meta-analysis, as indicated by the funnel plot (Figure 4) and Egger test (*p* = 0.33), and the overall effect size remained significant (OR = 1.34; 95% CI, 1.21–1.48) after trim-and-fill correction for missing data.

**TABLE 3:** Results of grading of recommendations, assessment, development, and evaluations (GRADE) analysis.

Certainty assessment							Effect		Certainty	Importance
No. of studies	Study design	Risk of bias	Inconsistency	Indirectness	Imprecision	Other considerations	Relative (95% CI)	Absolute (95% CI)		
Risk of any fracture										
10	Observational studies	Serious	Serious	Not serious	Not serious	Dose-response gradient	OR 1.39 (1.26, 1.52)	1 fewer per 1,000 (from 2 fewer to 1 fewer)	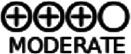	Critical
Risk of fracture requiring hospitalization										
4	Observational studies	Serious	Serious	Not serious	Not serious	None	OR 1.55 (1.29, 1.88)	2 fewer per 1,000 (from 2 fewer to 1 fewer)	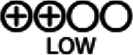	Critical

## Discussion

A previous meta-analysis reported that ADT is associated with decreased BMD (Kim et al., 2019) and elevated fracture risk (Serpa Neto et al., 2010) among prostate cancer patients. In the present meta-analysis based on cohort studies, ADT was associated with increased risks of fracture and fracture requiring hospitalization. Furthermore, consistent trends were observed for various disease conditions, dosage levels, treatment regimens, fracture sites, and age groups, with varying levels of heterogeneity. The certainty of evidence regarding the risks of any fracture and fracture requiring hospitalization was moderate and low, respectively.

The pathophysiology of prostate cancer relies on androgen and corresponding receptor signaling (A M El Batri et al., 2019), and the mechanism underlying ADT-induced fracture primarily involves a marked increase in bone turnover and alteration of the fat to lean body mass ratio (Santini et al., 2020). During adaptation to stress, constant turnover and remodeling occur and are processed through numerous transmitters, including osteoblasts, osteoclasts, hormones, and other related factors. Moreover, receptor activator of nuclear factor-κB ligand plays an important role in osteoclast activity, including differentiation (Bienz and Saad, 2015). Furthermore, ADT is potentially associated with modifications in body composition that lead to obesity (Santini et al., 2020). Obesity has a detrimental effect on bone health through hormonal dysregulation, oxidative stress, and inflammation. Sarcopenia induced by an increase in fat body mass directly and negatively affects skeletal structure, posing an additional threat to bone health (Santini et al., 2020).

The present stratified analysis revealed that both localized and unknown stage prostate cancer are associated with an increased risk of fracture. For localized prostate cancer, long-term ADT is recommended along with salvage treatment after RP for high-risk patients. Furthermore, short-term and long-term ADT are recommended after RT. By contrast, evidence regarding the optimal duration for neoadjuvant ADT and prostate-specific antigen intervention for salvage treatment is lacking (Zhou, 2019). In this study, ADT was associated with increased risks of any fracture and fracture requiring hospitalization. ADT remains the gold standard treatment for metastatic prostate cancer (NCCN, 2020). Although the exact mechanism underlying bone metastasis in prostate cancer remains unclear, the bone microenvironment is considered the key mediator of the tropism facilitating cancer cell migration to the bones through a cytokine gradient (A M El Batri et al., 2019). Patients with metastatic prostate cancer are at a much higher risk of skeletal events during ADT (Hussain et al., 2016). The inherent pathophysiology of metastasis and pharmacological properties of ADT might increase the risk of fracture among specific patients regardless of the primary treatment.

All types of ADT were associated with an elevated risk of fracture in this study. LHRH agonists have been considered the first-line treatment for almost all stages and grades of prostate cancer, including in patients considered at risk for fracture (Moreau et al., 2006). Antiandrogen, which poses a nonsignificant elevation in the risk of fracture requiring hospitalization, competes with endogenous androgen to bind to androgen receptors rather than eliminating circulating androgens, thus, androgen suppression is limited (Ricci et al., 2014). Increased estrogen level and loss of lean mass muscle mitigation derived from antiandrogen might also contribute to BMD preservation superior to LHRH agonist (Smith et al., 2004). Because androgen monotherapy has limited efficacy and a low survival rate (Seidenfeld et al., 2000),current guidelines have demoted it to the combine treatment strategy or to palliative therapy for patients with advanced or metastatic disease (NCCN, 2020). These confounding factors may partially explain the different risks of fracture associated with LHRH agonists and antiandrogen.

Three studies evaluated the effect of dose on the risk of fracture (Shao et al., 2013; Wu et al., 2015; Nguyen et al., 2018), and the methods of dosage selection remain similar to those reported by Shahinian et al. (2005). Nguyen et al. (2018) has been excluded from the analysis due to the shorter research period compared with Shao et al. (2013). Furthermore, the dosage-related risk was identified, but the long-term risk can only be detected through cohort studies as supplemental information not mentioned in clinical trials. Shao et al. (2013) investigated ADT as primary and adjunctive therapy and the risk of fracture with increased baseline risk of skeletal complications; Wu et al. (2015) and Nguyen et al. (2018) did not perform subgroup analysis, thus limiting interpretation of the different levels of fracture risk among various ADT patterns.

Age remains a crucial risk factor for fracture, and age was adjusted for in all included studies. Prespecified analysis revealed that bone loss among the general population individuals receiving ADT is more rapid and severe than that among elder individuals, and BMD reduction, and thus increased fracture risk, was observed at multiple skeletal sites (Eastham, 2007). The present stratified analysis found that the risk of fracture among patients aged >65 years was lower than that among the general population; ADT was found to increase the risk of fracture among all age groups, without significant changes in heterogeneity, after stratified and sensitivity analyses. Heterogeneity might be partially reflected the complicated mechanism.

This study has several limitations. First, information regarding lifestyle-related (calcium/vitamin D intake, nutrition status, exercise intensity and frequency, and history of fracture) and disease-related (cancer risk groups, genetic composition, and family history) factors, RT dose and RP, the purpose of ADT intervention (neoadjuvant, adjuvant, or salvage treatment), and antiresorptive medication use were not well documented in every study. In addition, most cohort studies did not adequately indicate the RT dose or divided radical surgery into different groups because of database limitations, potentially resulting in heterogeneity in the results, even after sensitivity analysis. Second, after RP, patients receiving ADT for localized disease were prone to disease progression to the advanced stage, biochemical failure, and micrometastasis, potentially resulting in a selection bias. Therefore, the certainty of evidence remains low to moderate because of the risk of bias and inconsistency among the included studies. Finally, data on fractures treated with LHRH antagonists and second-generation antiandrogen remain scarce. Further studies are required to confirm the differential risks of these agents on fracture.

## Conclusion

This study reports that ADT is significantly associated with an elevated risk of fracture among patients with localized and unknown stage prostate cancer. The elevated risk is positively correlated with the duration of ADT. Furthermore, analyses stratified by age and ADT regimen revealed similar risks. However, different treatment indications and disease populations potentially contribute to significant heterogeneity in outcomes. Well-designed prospective trials with intact information of risk factors on fracture are warranted to overcome these limitations.

## Data Availability

The original contributions presented in the study are included in the article/Supplementary Material, further inquiries can be directed to the corresponding author.
