# Supplementary material for: Risk of Fracture During Androgen Deprivation Therapy Among Patients With Prostate Cancer: A Systematic Review and Meta-Analysis of Cohort Studies
Source: Front Pharmacol. 2021 Aug 6;12:652979. doi: 10.3389/fphar.2021.652979 (PMC8378175; doi:10.3389/fphar.2021.652979)
Supplement: Supplementary file 1 [file DataSheet5.doc]

**Supplemental material 5.** Methodological Quality of Included Cohort Studies

| Study | Selection | Score | Comparability | Score | Outcome | Score | Total score |
| --- | --- | --- | --- | --- | --- | --- | --- |
| M. Wallander et al, 2019, Sweden | Representative prostate cancer population between 1987 and 2014; unexposed according to the  Drug Dispensation Register as exposure;  history of previous fracture as a risk factor | 3 | Study controlled for  physiological parameters (age, height, weight), disease condition (previous fracture, rheumatoid arthritis, secondary osteoporosis, CCI, previous known fall),  drug use (glucocorticoid use, alendronate, calcium + vitamin D) and substance use history (estimated overconsumption of alcohol) | 2 | Diagnosis by *ICD* code;  follow-up time: 0.8-1.3 years (adequate);  reason for incomplete follow-up  was death (4086) in the prostate cancer and ADT group (no information on other attrition causes) | 2 | 7 |
| Chi Nguyen et al, 2018, USA | SEER-Medicare-linked database (>66 y); unexposed population from same database;  Healthcare Common Procedure Coding System used as exposure assessment | 4 | Propensity score–matched retrospective cohort (adjusted for patient demographic and tumor characteristics) | 2 | Diagnosis by *ICD* code;  cumulative incidence included for 3, 5, and 10 years;  propensity-score matching for 58 981 patients in ADT and non-ADT groups | 3 | 9 |
| Chi-Ho Lee et al, 2017, China | Single-center database; no unexposed cohort; exposure determined from record in hospital database; patient excluded if they had previously documented osteoporosis or fracture | 2 | Multivariable Cox regression analysis controlled for age, smoking,  comorbidities (DM, HTN, hypercholesterolemia, CAD, stroke), medications (steroid, Ca++, vitamin D, antiosteoporosis medication) and ADT | 2 | Diagnosis by *ICD* code; median follow-up of 5 years; lack of information on adequacy of follow-up cohort | 2 | 6 |
| Christopher J. D. Wallis et al, 2016, Canada | SEER-Medicare-linked database (>66 y); unexposed population from same database; Healthcare Common Procedure Coding System as exposure assessment; skeletal complications included history of osteoporosis as a covariate | 3 | Cox proportional  hazards regression controlled for age, grade, race, marital status, CCI, and pre-diagnosis history of osteoporosis | 2 | Diagnosis by *ICD* code; median follow-up of 5 years; figures regarding cohort selection included, including inclusion/exclusion criteria and causes of ineligibility | 3 | 8 |
| C.-T. Wu et al, 2015, Taiwan | National Health Insurance Research Database population-based data from Taiwan; unexposed from same cohort; Anatomical Therapeutic Chemical code as exposure assessment; patients with history of fractures or within 12 mo of diagnosis of prostate cancer were excluded | 4 | Cox proportional hazards regression controlled for age, socioeconomic status, CCI, and other cancer treatments received | 2 | Diagnosis by *ICD* code; only patients who survived ≥5 years were enrolled; only patients with complete follow-up were enrolled | 3 | 9 |
| Alice Wang et al, 2015, New Zealand | New Zealand Cancer Registry; unexposed population from same database; Pharmaceutical Collection;  not mentioned whether patients with fracture history were included | 3 | Binary logistic regression controlled for age and ethnicity only | 1 | Diagnosis by *ICD* code; follow-up time not mentioned; only patients with complete follow-up were enrolled | 2 | 6 |
| Jeremy Yuen Chun Teoh et al, 2015, China | Single-hospital database; unexposed population from same database; exposure determined from hospital database; not mentioned whether patients with fracture history were included | 2 | Cox proportional  hazards regression controlled for age, DM, HTN, hyperlipidemia, preexisting ischemic heart disease, ECOG performance status, and ADT;  few factors were controlled | 1 | Diagnosis from hospital database; optimal follow-up time (mean: 72.9 months); only patients with complete follow-up were enrolled | 3 | 6 |
| AK Morgans et al, 2014, USA | Prostate Cancer Outcomes Study (SEER database); unexposed population from same database; self-reported ADT at 6 mo and 1, 2, 5, and 15 y after diagnosis; not mentioned whether patients with fracture history were included | 2 | Weighted logistic regression controlled for age, ADT duration, race, marital status, CCI, and Gleason score | 2 | Diagnosis from self-reported fracture; optimal follow-up time (15 years); only patients with complete follow-up were enrolled | 2 | 6 |
| Antti Kaipia et al, 2014, Finland | Tampere University Central Hospital  Records; unexposed population from same database; exposure determined from hospital database; not mentioned whether patients with fracture history were included | 2 | Binary logistic regression adjusted for age and PSA level | 1 | Diagnosis by *ICD* code and experienced orthopedic surgeon for classification of hip fracture; optimal follow-up time (mean: 4.23 years); 1 patient excluded (missing data) | 3 | 6 |
| Yu-Hsuan Shao et al, 2013, USA | SEER-Medicare-linked database (>66 y); unexposed population from same database; Healthcare Common Procedure Coding System as exposure assessment; patients with any fracture that occurred before ADT or within 1 y of diagnosis were not included | 4 | Cox proportional hazards regression controlled for age, diagnosis year, race, education, income, cancer grade, cancer stage, comorbidity, bisphosphonate therapy, and cumulative dose of ADT | 2 | Diagnosis by *ICD* code; optimal follow-up time (12 years); only patients with complete follow-up were enrolled | 3 | 9 |
| Jennifer L. Beebe-Dimmer et al, 2012, USA | SEER-Medicare-linked database (>66 y); unexposed population from same database; Healthcare Common Procedure Coding System as exposure assessment; history of fracture adjusted for in final model | 3 | Cox proportional hazards regression controlled for age, race, tumor grade, clinical T stage, comorbidity, history of fracture, osteoporosis or osteopenia prior to prostate cancer diagnosis, and primary treatment | 2 | Diagnosis by *ICD* code; optimal follow-up time (3-10 years); only patients with complete follow-up were enrolled | 3 | 8 |
| Shabbir M. H. Alibhai et al, 2010, Canada | Administrative data from the Institute for Clinical Evaluative Sciences; unexposed population from same database; Ontario Drug Benefit Formulary used for exposure assessment; population with history of fracture included | 3 | Cox proportional hazards regression using 1:1 propensity-score matching for age, ADT, prior bone thinning medication, prior chronic kidney disease, prior dementia, prior fragility fracture, prior osteoporosis diagnosis or treatment, prior rheumatological disease, and regular primary care access | 2 | Diagnosis by *ICD* code; optimal follow-up time (mean: 6.47 years); only patients with complete follow-up were enrolled | 3 | 8 |
| Matthew R. Smith et al, 2006, USA | Database of medical claims from 16 large American companies; unexposed population from same database; pharmacy claims (national drug codes) used as exposure assessment; unclear delineation of history of fracture | 3 | Multivariate logistic regression controlled for age, LHRH agonist treatment, comorbidity, income, observation period, geographic region, and health plan | 2 | Diagnosis by *ICD* code; short follow-up time (20.5-22.1 months); only patients with complete follow-up were enrolled | 2 | 7 |
| Matthew R. Smith et al, 2005, USA | Medicare Public Use File databases (Medicare beneﬁciaries); unexposed population from the same database;  Medicare codes J9217, J9218, J9202, and J1950 used as exposure assessment; unclear delineation of history of fracture | 3 | Cox proportional hazards regression controlled for age, race, geographic location, cardiovascular disease, DM, LHRH agonist treatment, and duration of LHRH agonist treatment | 2 | Diagnosis by *ICD* code; optimal follow-up time (7-9 years); only patients with complete follow-up were enrolled | 3 | 8 |
| Vahakn B. Shahinian et al, 2005, USA | SEER-Medicare linked database (>66 y); unexposed population from same database; Healthcare Common Procedure Coding System as exposure assessment; patients with history of fractures excluded | 4 | Cox proportional hazards regression controlled for age; ADT; race; SEER region; cancer grade; cancer stage; year of diagnosis; level of education; income; CCI; number of provider visits within the 12 months before diagnosis; presence of osteoporosis, osteopenia, or fracture within the 12 months before diagnosis; and treatment with RP or RT | 2 | Diagnosis by *ICD* code; optimal follow-up time (7-9 years); only patients with complete follow-up were enrolled | 3 | 9 |
| Ana M. López et al, 2005, Spain | Hospital database; unexposed population from same database, pharmacy registries; history of fracture included (adjusted for in the model) | 3 | Cox proportional hazards regression controlled for age, previous fracture, smoking, alcohol, and ADT | 1 | Hospital charts; optimal follow-up time (48-68 months); only patients with complete follow-up were enrolled | 2 | 6 |

Abbreviations: ADT, androgen deprivation therapy; CAD, coronary artery disease; CCI, Charlson comorbidity index; DM, diabetes mellitus; HTN, hypertension; *ICD*, *International Classification of Diseases*; LHRH, luteinizing hormone–releasing hormone; PSA, prostate-specific antigen; RP, radical prostatectomy; RT, radiotherapy; SEER, Surveillance, Epidemiology, and End Results.
