# Supplementary figures and images for: Risk of Fracture During Androgen Deprivation Therapy Among Patients With Prostate Cancer: A Systematic Review and Meta-Analysis of Cohort Studies
Source: Front Pharmacol. 2021 Aug 6;12:652979. doi: 10.3389/fphar.2021.652979 (PMC8378175; doi:10.3389/fphar.2021.652979)

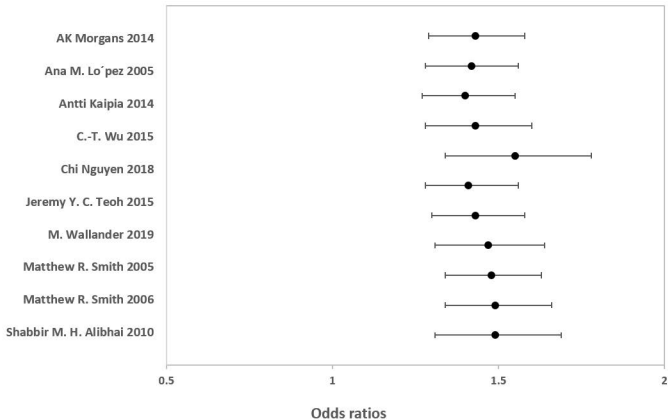

Supplement: Supplementary file 2 [file DataSheet4.pdf]
