# Supplementary material for: Risk of Fracture During Androgen Deprivation Therapy Among Patients With Prostate Cancer: A Systematic Review and Meta-Analysis of Cohort Studies
Source: Front Pharmacol. 2021 Aug 6;12:652979. doi: 10.3389/fphar.2021.652979 (PMC8378175; doi:10.3389/fphar.2021.652979)
Supplement: Supplementary file 4 [file DataSheet3.doc]

**Supplemental material 3. Searching Strategy**

Relevant articles, published before March 31, 2020, were retrieved from 3 electronic databases (Cochrane Library ([in Wiley]), PubMed, and Embase (embase.com). The following search terms were used with the language restricted to English:

**Cochrane Central**

#1 “prostate cancer”

#2 “androgen deprivation OR androgen suppression OR chemical castration OR Antiandrogen OR Gonadotropin releasing hormone agonist”

#3 “fracture”

#4 #1 AND #2 AND #3

**Embase.com**

(“prostate cancer” AND “androgen deprivation” OR (“androgen” AND “suppression”) OR “chemical castration” OR “antiandrogen” OR “gonadorelin agonist”) AND “fracture”

**PubMed**

("prostatic neoplasms"[MeSH Terms] OR ("prostatic"[All Fields] AND "neoplasms"[All Fields]) OR "prostatic neoplasms"[All Fields] OR ("prostate"[All Fields] AND "cancer"[All Fields]) OR "prostate cancer"[All Fields]) AND ((("androgens"[Pharmacological Action] OR "androgens"[MeSH Terms] OR "androgens"[All Fields] OR "androgen"[All Fields]) AND deprivation[All Fields]) OR (("androgens"[Pharmacological Action] OR "androgens"[MeSH Terms] OR "androgens"[All Fields] OR "androgen"[All Fields]) AND suppression[All Fields]) OR (("J Mol Catal A Chem"[Journal] OR "chemical"[All Fields]) AND ("orchiectomy"[MeSH Terms] OR "orchiectomy"[All Fields] OR "castration"[All Fields] OR "castration"[MeSH Terms])) OR ("androgen antagonists"[Pharmacological Action] OR "androgen antagonists"[MeSH Terms] OR ("androgen"[All Fields] AND "antagonists"[All Fields]) OR "androgen antagonists"[All Fields] OR "antiandrogen"[All Fields]) OR (("gonadotropin-releasing hormone"[MeSH Terms] OR ("gonadotropin-releasing"[All Fields] AND "hormone"[All Fields]) OR "gonadotropin-releasing hormone"[All Fields] OR ("gonadotropin"[All Fields] AND "releasing"[All Fields] AND "hormone"[All Fields]) OR "gonadotropin releasing hormone"[All Fields]) AND agonist[All Fields])) AND ("fractures, bone"[MeSH Terms] OR ("fractures"[All Fields] AND "bone"[All Fields]) OR "bone fractures"[All Fields] OR "fracture"[All Fields]) AND English[lang]
