# Supplementary material for: Risk of Fracture During Androgen Deprivation Therapy Among Patients With Prostate Cancer: A Systematic Review and Meta-Analysis of Cohort Studies
Source: Front Pharmacol. 2021 Aug 6;12:652979. doi: 10.3389/fphar.2021.652979 (PMC8378175; doi:10.3389/fphar.2021.652979)
Supplement: Supplementary file 5 [file DataSheet2.doc]

**Supplemental material 2. Meta-analysis of Observational Studies in Epidemiology (MOOSE) Checklist**

| Criterion | | Brief description of how the criterion was handled in the meta-analysis |
| --- | --- | --- |
| Reporting of background should include | |  |
| √ | Problem definition | Androgen deprivation therapy (ADT) is the first-line treatment strategy for prostate cancer. However, various adverse effects of ADT warrant further assessment. ADT-related fracture is characterized by increased bone turnover and altered fat to lean body mass ratio. Meta-analysis and stratified analyses on fracture risk and ADT remain insufficient. |
| √ | Hypothesis statement | ADT poses a risk of fracture through androgen blockade. |
| √ | Description of study outcomes | We primarily analyzed the risk of fracture from ADT and performed several stratified analyses, including by disease condition, treatment regimen, dose level, age, and fracture site. |
| √ | Type of exposure or intervention used | ADT |
| √ | Type of study design used | Cohort studies |
| √ | Study population | Prostate cancer patients were included in this study. Exclusion criteria were focuses on changes in bone mineral density or the risk of osteoporosis. Articles lacking, including narrative reviews, case reports, case series, editorials, letters, guidelines, treatment consensus, and conference abstracts, lacking data were excluded. |
| Reporting of search strategy should include | |  |
| √ | Qualifications of searchers | The credentials of the investigators are indicated in the authors list, including “pharmacist,” “clinician,” and “professors.” |
| √ | Search strategy, including time period included in the synthesis and keywords | The search strategy is explained on page 9 of the manuscript and detailed in figure 1.  The study periods are detailed on page 5 of the manuscript. |
| √ | Databases and registries searched | Cochrane Library, PubMed, and EMBASE. |
| √ | Search software used, name and version, including special features | Search software was not used.  EndNote was used for inserting citations, eliminating duplications, and conducting stratified analyses. |
| √ | Use of hand searching | Manual bibliography search was employed to identify additional references. |
| √ | List of citations located and those excluded, including justifications | Details of the search process and justification of inclusion and exclusion are provided in figure 1. |
| √ | Method of addressing articles published in languages other than English | We applied a language restriction to obtain articles only in English. |
| √ | Method of handling abstracts and unpublished studies | Conference abstracts belonged were excluded. |
| √ | Description of any contact with authors | Authors of the included studies were contacted if any data were missing or insufficient for the present analyses. |
| Reporting of methods should include | |  |
| √ | Description of relevance or appropriateness of studies assembled for assessing the hypothesis to be tested | Inclusion and exclusion criteria are described in the methods section. |
| √ | Rationale for the selection and coding of data | A self-developed data recording form was applied for data coding of the relevant information, including the individual authors, years of publication, age of the included population, study size, study source, exposure assessment, radiotherapy and radical prostatectomy documentation, categories of ADT, antiresorptive medication use, outcome categories, fracture definitions, adjusted factors, and conflicts of interest |
| √ | Assessment of confounding | Potential confounding factors derived from separate studies are outlined in table 1. Furthermore, sensitivity analyses were performed to assess the effects of different confounding factors. |
| √ | Assessment of study quality, including blinding of quality assessors; stratification or regression on possible predictors of study results | The Newcastle-Ottawa Scale (NOS) was used to evaluate the quality of eligible cohort studies included in the present review. |
| √ | Assessment of heterogeneity | *I*^2^ was used to evaluate statistical heterogeneity, and clinical heterogeneity was further surveyed through stratified analyses. |
| √ | Description of statistical methods in sufficient detail to be replicated | Analyses were performed using Review Manager software, version 5.4.1 (Cochrane) using a random-effects model. Each effect size and its corresponding 95% confidence intervals (CI) were extracted. All *P* values were 2-sided, with statistical signiﬁcance set at *P* < .05. |
| √ | Provision of appropriate tables and graphics | We included 5 figures, 4 tables, and 3 supplemental tables for this review. |
| Reporting of results should include | |  |
| √ | Graph summarizing individual study estimates and overall estimate | Figure 2 & eFigure 1 |
| √ | Table giving descriptive information for each study included | Table 1 |
| √ | Results of sensitivity testing | The results of sensitivity analyses are presented in Table 2. |
| √ | Indication of statistical uncertainty of findings | The 95% CI are presented if available. |
| Reporting of discussion should include | |  |
| √ | Quantitative assessment of bias | We focused on the results of stratified analyses and attempted to determine the effects on potential bias. |
| √ | Justification for exclusion | Justification for exclusion is included on page 12-13 of the manuscript and flowchart in Figure 1. |
| √ | Assessment of quality of included studies | The NOS was used for quality evaluation of the cohort studies included in this review. |
| Reporting of conclusions should include | |  |
| √ | Consideration of alternative explanations for observed results | ADT was associated with an elevated fracture risk. Several stratified analyses and sensitivity analyses revealed consistent trends in the risk estimates. |
| √ | Generalization of the conclusions | Our analyses can reinforce the pharmacologic mechanism of ADT and corresponding fracture risk. Furthermore, stratified analyses of disease condition, dose level, ADT regimen, and fracture site indicated similar trends with different risk estimates that may facilitate clinical decision making. |
| √ | Guidelines for future research | Complex interactions between risk factors for fracture may contribute to significant heterogeneity, and comprehensive records regarding key factors associated with prostate cancer are significant for further research. Well-designed randomized controlled trials are warranted to overcome this limitation. |
| √ | Disclosure of funding source | This study did not receive any funding. |
